# Efficacy of Chemical and Biological Stump Treatments for the Control of *Heterobasidion occidentale* Infection of California *Abies concolor*

**DOI:** 10.3390/pathogens10111390

**Published:** 2021-10-27

**Authors:** Adrian L. Poloni, Matteo Garbelotto, Christopher A. Lee, Richard C. Cobb

**Affiliations:** 1 Department of Natural Resources & Environmental Science, California Polytechnic State University, San Luis Obispo, CA 93407, USA; adrian_poloni@live.com; 2 Department of Environmental Science, Policy, and Management, University of California, 137 Mulford Hall, Berkeley, CA 94720, USA; matteog@berkeley.edu; 3 California Department of Forestry and Fire Protection, 118 S. Fortuna Blvd., Fortuna, CA 95540, USA; christopher.lee@fire.ca.gov

**Keywords:** Heterobasidion root disease, *Phlebiopsis gigantea*, white fir, bark beetle, California

## Abstract

We conducted an experimental evaluation of treatments to limit *Heterobasidion occidentale* infection of white fir (*Abies concolor*) stumps and wounds in California mixed conifer forests. We tested the efficacy of urea, borate, and a mixture of two locally collected *Phlebiopsis gigantea* strains in preventing pathogen colonization of fir stumps and separately, urea and borate as infection controls on experimental stem wounds. These were paired with a laboratory test on ~100 g wood blocks with and without a one-week delay between inoculation and treatment. Urea, borates, and *Phlebiopsis* treatments all significantly reduced the stump surface area that was colonized by *H. occidentale* at 84%, 91%, and 68%, respectively, relative to the controls. However, only the borate treatments significantly lowered the number of stumps that were infected by the pathogen. The laboratory study matched the patterns that were found in the stump experiment with a reduced area of colonization for urea, borates, or *P. gigantea* treatments relative to the controls; delaying the treatment did not affect efficacy. The field wound experiment did not result in any *Heterobasidion* colonization, even in positive control treatments, rendering the experiment uninformative. Our study suggests treatments that are known to limit *Heterobasidion* establishment on pine or spruce stumps elsewhere in the world may also be effective on true firs in California.

## 1. Introduction

*Heterobasidion* species occur across the northern hemisphere and severely impact a range of conifers including, but not limited to, the widespread economically and ecologically relevant genera *Pinus*, *Abies*, and *Picea* [1]. These pathogens are largely, but not exclusively, endemic causes of Heterobasidion root disease, a root and butt rot disease which is one of the most important diseases impacting conifers in the northern hemisphere [2,3]. Since native root pathogens play important roles as engineers and regulators of forest structure, it is likely that shifts in pathogen prevalence, host distribution, or host mortality will impact additional forest management problems including fire risk, insect outbreak, and sustained ecosystem functions including water and carbon cycling [3,4,5]. Identifying management options that can limit not only root disease establishment and/or impacts but also the development of butt rots that affect tree vigor could be a worthwhile effort to sustain the health of forest resources in much of the northern hemisphere.

White fir (*Abies concolor*) and red fir (*Abies magnifica*) collectively are widespread across much of the western United States (US). White fir in particular is one of the most common, and often dominant trees, across the Sierra Nevada and Cascade mountain ranges of California [6,7]. As is the case for similar forests across the western US, these forests have recently experienced high levels of mortality that are associated with multiple disturbance agents [8,9,10]. *Heterobasidion occidentale*, other native root diseases, *Scolytus ventralis* (the fir engraver beetle), and drought have each been important sources of landscape-level mortality for these important forest trees [9,11,12]. Two *Heterobasidion* pathogens are widespread in the western US: *H. occidentale* and *H. irregulare* which primarily attack *Abies* and *Pinus* species, respectively [13]. Each pathogen causes disease which can persist for decades in a diverse set of disturbance histories, host abundance, and environmental conditions [14,15,16]. Along with substantial local mortality, *H. occidentale* infection appears to reduce tree vigor which creates the potential for interaction with other biotic and abiotic disturbances [15,17,18]. Thus, management of forests with fir components may benefit from actions that are meant to limit infection of stumps and trees by *H. occidentale*.

Primary infection by *H. occidentale* basidiospores may occur on freshly cut stumps and on fresh wounds that are often, although not exclusively, associated with forest harvest [19,20]. The subsequent disease development in neighboring trees occurs either via secondary fungal growth through root–root contacts or by priority effects that are triggered by a diseased tree on its neighbors [3,14,20]. *Heterobasidion* growth rate tends to slow after what can be a locally intense and rapid spread for the first 10 years following disease emergence, however, disease centers can also create canopy openings which can persist for decades [15,21]. *Heterobasidion occidentale* can infect both the heartwood and the sapwood of true firs [14], causing not only a significant reduction of timber value, loss of tree stability, but also decreased tree vigor and growth.

*Heterobasidion occidentale* root and butt disease is widespread in western forests, but practical treatments to prevent infection have not been developed for this region. A well-developed body of literature suggests that treatments that were designed and tested for other *Heterobasidion*—host systems may also apply to the *H. occidentale*—white fir host system using relatively affordable and environmentally benign treatments [22,23,24]. Specifically, a clear and growing number of studies, largely performed outside California, indicates that stump treatment during logging operations can limit pathogen establishment [22,25,26]. Infection by *Heterobasidion* spp. has been effectively managed in the southeastern US, Canada, and throughout Europe by applying treatments to stump surfaces. These prevention treatments typically apply chemicals including borates, urea, or *Phlebiopsis gigantea* (a widespread saprotroph which can outcompete *Heterobasidion* species on stumps), thus acting as a biocontrol [22,24,27,28]. *Phlebiopsis gigantea* biocontrol formulations are particularly promising in that they may be active much longer than chemical treatments, and thus provide longer-term protection without the obvious side effects of chemical treatments [29]. Although borates have been used in the western US to control Heterobasidion disease in pines, biocontrol agents have never been tested on western pines or other susceptible western US tree species. In particular no stump or wound treatments, biological or chemical, have ever been tested to control Heterobasidion root and butt rot disease in true firs within the US, including western US forests. Although some forest soils have been shown to be suppressive against *Heterobasidion* spp. [30] and to affect the efficacy of treatments [31], this is not the case for the sandy-loam soils that are typical of both mixed conifer and pine forests in the Western US. These soils are susceptible to summer drying and generally regarded as conducive to disease establishment and spread [32].

*Heterobasidion* inoculum appears to be capable of persisting in a forest stand for at least ~70 years. Thus, once established in a site, the pathogen is likely to cause disease in more than one tree generation [3,17]. Even when direct tree mortality does not occur, trees that are infected by *Heterobasidion* spp. are predisposed to mortality due to other causes, both biotic and abiotic which may occur over stand–landscape scales [14,16,17]. Landscape tree mortality and forest decline is an increasing management challenge, and indeed these large scale events appear to emerge from multiple and often interacting disturbances [33,34]. Many studies have documented insect colonization after wildfire and drought [35,36,37], and some studies have suggested that there are positive associations between root disease and insect attack [15,38]. Given the long-term dynamics of *H. occidentale* in these forests, it is certain that root disease and bark beetle colonization will frequently overlap, if not directly interact (Rizzo and Slaughter 2000).

In this study we aimed to accelerate the development of treatments for *H. occidentale* in the Sierra Nevada and Southern Cascade mountains to both prevent long-term and persistent disease impacts as well as mitigate any interactions with native bark beetles. We evaluated the effectiveness of two chemical treatments (urea and borate) and one biological control (*P. gigantea*) treatment in preventing *Heterobasidion* establishment on recently cut stumps and the effectiveness of two chemical treatments (urea and borate) in preventing *Heterobasidion* establishment on wounds that were associated with forest management operations. We evaluated these treatments in an actively managed timber production system within the Sierra Nevada range which is representative of many timber production scenarios throughout the region. First, we determined if borate, urea, and *P. gigantea* topical treatments to white fir stumps could effectively control *H. occidentale* establishment and growth. Second, we applied urea and borate on the wounds to estimate their efficacy in preventing pathogen establishment on tree wounds. While studying the efficacy of this treatment, we also monitored landing rates of *S. ventralis* to test for potential *Heterobasidion*–bark beetle associations. Lastly, the same treatments that were applied in the stump study were replicated on wood blocks in the laboratory. Previous field experiments suggested that we would observe reduced pathogen colonization in stumps and wounds for each treatment type. We understand this trial to be unprecedented given that, to our knowledge, all available data have been generated for tree species other than white fir and through experiments that, for the most part, did not include western U.S. forests.

## 2. Methods

### 2.1. Study Site

Our stump and wound experiments were conducted at the Blodgett Forest Research Station (Blodgett Forest), located on the western slope of the north-central Sierra Nevada mountain range, near Georgetown, California (38°52′ N; 120°40′ W; 1220 to 1310 m elevation). The 1214 ha forest has been actively managed, employing a broad array of silvicultural treatments including clear-cut, partial cutting, uneven-aged, and even-aged management of white fir. Blodgett forest is representative of the mixed-conifer forest that is present in much of the western Sierra Nevada and includes substantial amounts of sugar pine (*Pinus lambertiana*), ponderosa pine (*Pinus ponderosa*), white fir, incense-cedar (*Calocedrus decurrens*), and Douglas-fir (*Pseudotsuga menziesii*). Broad leaved species such as California black oak (*Quercus kelloggii*), tanoak (*Notholithocarpus densiflorus*), and Pacific madrone (*Arbutus menziesii*) are also present, although less dominant than conifers.

Heterobasidion root disease is endemic and widespread in this regional forest type [20,39]. Since *Heterobasidion* spp. inoculum varies greatly in time and space [40,41], we also monitored *Heterobasidion* airborne inoculum at each experimental site on the same day that the treatments were applied in October 2019 to improve the comparability across the sites and ensure that the differences between the treatments were not associated with variation in ambient inoculum. We intentionally timed the treatments to overlap with the expected period of the greatest ambient inoculum [41,42] but recognized that this must nonetheless be measured. Airborne inoculum was estimated with the wood disc exposure method of Gontheir et al. [43], modified as follows. We measured the ambient inoculum on 20, 9–13 cm diameter wood discs (de-barked *A. concolor*) for 24 h at each stump or wounding experiment location, at a total of five stands (Blodgett Forest “compartments”) which were used for the stump and wound experiments (two stands) or for the wounding experiment (three stands). Individual measurements were grouped into five sets of four discs that were distributed as a cross within each site, with each set of four separated by 50 m. This resulted in 100 total measurements of ambient inoculum at 5 independent stands that were separated by at least 500 m. All wood discs were treated with a benomyl solution (20 mg L^−1^) immediately after sampling and kept at ~5 °C until they reached the laboratory at U.C. Berkeley. At U.C. Berkeley, discs were kept at room temperature and inspected for the presence of colonies of the *Spiniger* imperfect state of the fungus after 9, 14, and 21 days. Colonies were marked and counted, and three colonies per disc were sub-cultured on 30% malt extract agar (MEA) plates using a sterile scalpel. We used DNA that was collected from each colony as an identification method for *Heterobasidion* species via a PCR analysis which employed *Heterobasidion*-specific primers and an established laboratory protocol [44].

### 2.2. Field Experiments

We identified trees for the stump control experiment June and July 2019 in two stands (compartments 310 and 670) where forest operations were scheduled two months later. Each stand was selected due to the apparent absence of Heterobasidion root disease centers and on the basis of a similar soil type, mostly a loamy Alfisol with a well-developed O layer. These criteria allowed our study to inform the infection prevention efforts in forests where inoculum was known to be abundant, soils that were conducive to the disease, but the disease had not yet emerged at a particular location. One study site, compartment 310, was further divided into two sections, a selective harvest (some canopy retention) and a clear-cut harvest via operational plans that were conceived and executed independently of our study. Each tree that was selected for the stump *Heterobasidion* prevention experiment was measured for diameter at breast height (dbh ~1.36 m height) and tree base (stump) diameter, rated for overall health, tagged, and other pest and pathogen signs recorded. Trees with any symptoms of possible *Heterobasidion* infection, such as butt or heart rot were excluded, including symptoms that were found after the initial harvest such as heartwood discoloration. Study trees were tagged at the base and marked for high stumping (~60 cm) by forestry crews. This also allowed the forestry operations to be fully complete before we conducted our prevention experiments, and allowed us to time the treatments according to the maximum expected ambient inoculum levels. Our experiment was completed within 80 days of forest harvest operations.

To establish the experiment, each stump was recut with a chainsaw to remove the top 25 cm and immediately treated with a manual application of one of four treatments, borate (12% *w*/*v* = 119.82 g/L; *n* = 30), urea (30% *w*/*v*, 300 g/L; *n* = 31), *P. gigantea* suspension (*n* = 34), or deionized water (control; *n* = 26). *Phlebiopsis* concentrations were in excess of 5.72 × 10^5^ colony forming units (CFU) mL^−1^ (see below for details). Each treatment was applied manually with a garden sprayer within 30–90 s of cutting until the entire stump surface was wet; approximately 1 L m^−2^ of solution was applied for each treatment. A total of 121 stumps were re-cut and treated across both compartments. Our *Phlebiopsis* suspension was produced in the laboratory from two isolates that were collected at Blodgett Forest. Approximately 5 L of solution was produced in the laboratory by growing cultures on 30% malt extract agar for 10–15 days and by gently scraping the surface of each plate after flooding with sterilized water. This solution was kept cool (3–5 °C), immediately measured for concentration through a series of dilutions, and applied to the study subjects within 20 days.

Approximately one year after treatment, each stump was revisited and photographed. The top 10 cm of each stump was debarked with a sterile hatchet and the entire debarked surface of the stump sprayed with 70% ethyl alcohol (ETOH). Using a chainsaw that was sterilized with 70% ETOH, the top 2–4 cm of each stump was cut and removed, and the stump was cut a second time to remove a 2–4 cm thick wood disc which was returned to the laboratory for evaluation of pathogen establishment, thus resulting in colonization estimates at ~5 cm depth within the stump. Within eight hours of sampling, all wood disc samples were treated with a benomyl solution (20 mg L^−1^) on all sides and then kept at ~5 °C during transport to UC Berkeley (within 24–48 h of sampling).

The wound experiment was replicated in five locations (compartments 270, 310, 420, 451, and 670) that encompassed a variety of previous management approaches including fir plantation silviculture and a range of selective harvest treatments. As in the stump experiment, we deliberately selected sites without obvious Heterobasidion root disease. Compartments were selected to be at distances of at least 500 m apart to capture the local differences in inoculum [40] and a wide range of tree sizes were included (25–82 cm dbh; see [24,40]). Trees were measured, marked, and selected with a similar procedure to that used in the stump experiment, but were additionally fitted with an adhesive panel trap (Elm beetle panel trap, Great Lakes IPM, 571 cm^2^) to monitor *S. ventralis* landings before and after wounding occurred on each tree (168 panels deployed). The panel traps were deployed twice, once from July 2019 to October 2019 (pre-treatment) and subsequently from July 2020 to October 2020 (post-treatment). Each panel was affixed on the north side of the bole at an average height of 139 cm. The fir engraver beetle has no aggregate pheromone but is known to colonize stressed trees [45,46], therefore we expected higher landing rates on trees with confirmed infections. Each study tree was revisited in October 2019 and an axe was used to create an artificial wound (average: 276.94 cm^2^) at ~1 m height on the north side of each bole. Each wound was immediately treated manually (typically within 30–90 s of wounding) using a garden sprayer to apply one of the four treatments, borate (12% *w*/*v*), urea (30% *w*/*v*), *H. occidentale* positive control, or deionized (DI) water (control), at the same application rate that was applied in the stump study (1 L m^−2^). A total of 183 trees were wounded and treated across the 5 sites with a total of 46 trees that were treated with borate solution, 46 treated with urea, 46 with the positive control treatment (*H. occidentale* inoculum solution), and 45 trees that were treated with sterile DI water (control). We applied each treatment on at least 10 trees at each site, with the exception of compartment 310 where we treated a total of 16 trees (4 per treatment type). Our *H. occidentale* suspension was produced in the laboratory from two isolates that were collected previously at Blodgett Forest with the wood disc trapping method. The same method for creating and measuring the spore suspension that was used for *Phlebiopsis* was repeated for *H. occidentale*: cultures were grown on 30% malt extract agar for 10–15 days, flooded with sterilized water, and gently scraped. This solution was kept cool, immediately measured for concentration through a series of dilutions, and applied to the study subjects within 20 days. The *H. occidentale* solution concentrations were consistently in excess of 6.91 × 10^5^ CFU mL^−1^.

One year after wounding and treatment, wood core samples were collected from 180 experimentally wounded white fir trees with 3 trees being excluded due to lost tree tags or because they had suffered subsequent wounds that were independent of our experiment. Using a sterilized Pulaski axe, 1–1.5 cm of surface was removed, exposing the surface of the wound. The wounds were sampled using a battery powered drill with a bit diameter and depth of 5.3 cm and 7 cm, respectively. The bit was sterilized between core samples which averaged 4.45 cm core depth. The cores were stored in an analogous matter to stump wood disc samples and all core samples from wounds and wood discs were transported from the field location to the UC Berkeley laboratory between 10 October and 31 October 2020.

### 2.3. Stump Experiment Sample Analysis

All of the wood disc samples were stored vertically in original plastic bags at (6 °C) for 3–5 days before systematic examination on day 3, 5, and 12 following sampling. Both sides of each wood disc were examined visually and using a mounted dissecting microscope at 10–30× magnification to identify *H. occidentale* colonies displaying the typical *Spiniger* conidiophores. The area of the disc that was covered by these colonies was delineated with a marker at each examination time. A sample of each colony per wood disc was obtained with a sterilized surgical needle to confirm the presence of *H. occidentale* establishment via direct culturing followed by PCR analysis performed on DNA that was extracted directly from fungal colonies growing on 30% MEA [44]. On the final examination (day 12), each wood disc with its delineated and confirmed colonies was photographed with a 30 cm long ruler as a reference. Each image was assessed to calculate the total pathogen coverage area in cm^2^. For each wood disc sample, we measured the total disc surface area and the area of the disk that was colonized by pathogen colonies. Only areas with fungal colonies that were present on both sides of each disc were measured, while colonies on a single side of the disc were regarded as contamination which occurred during the disc cutting and/or disc analysis. The two measurements, top and bottom, were averaged to estimate the total colonization. The treatment assignment was concealed from the surveyors that evaluated pathogen establishment.

### 2.4. Wound Experiment Sample Analysis

All wood core samples, in their individual bags, were kept refrigerated at 8–6 °C in a cold room until they were transferred for processing. Each core was cut in half with a band saw along its axis to produce two equal portions. Each portion and all sides (interior and exterior) of the wood core sample were examined visually and then using a microscope (10–30×) to identify the presence of *Heterobasidion* conidiophores. After each examination, the core was placed on a sterile paper plate, placed back into its original bag, and incubated at 25 °C. Each core was examined three times, at 3, 7, and 12 days after being cut into halves and the presence or absence of *Heterobasidion* conidiophores was recorded.

### 2.5. Laboratory Experiment and Sample Analysis

The wood block experiment was conducted in a growth chamber that was located on the campus of the California Polytechnic State University in San Luis Obispo, CA, USA. A set of large sample logs from recently cut (~1–4 days) white fir stumps were collected concurrently with stump treatments at Blodgett Forest in October 2019. The logs were immediately wrapped in plastic to prevent *H. occidentale* establishment from ambient inoculum and kept cool until they could be divided into 250 irregularly shaped wood blocks at the laboratory using a sterilized chainsaw and hatchet. A total of 96 wood blocks were cut, weighed, and then randomly assigned to one of eight treatments: borate, urea, *Phlebiopsis*, or sterile water (control) with or without a one-week treatment delay. We established 24 replicates for each treatment with half (12) assigned to the one-week delay treatment. Application rates and solution concentrations matched those of the stump experiment. Half of the blocks were first treated with chemical or biological control and then inoculum solution was applied one week later. The second set of blocks were first treated with *H. occidentale* inoculum (>6.91 × 10^5^ CFU mL^−1^ at 100 mL m^−2^), incubated for one week, and treatment was then applied. This procedure created a test of delayed, or post-inoculation treatment. Each block was assigned a unique number, measured to estimate the volume, and weighed at ambient moisture content prior to the beginning of the experiment. An additional set of 10 blocks were dried at 105 °C for 48 h to back estimate wood initial moisture content for the entire study. The wood block experiment was established in November 2019 and blocks were incubated at 20 °C for 11 months with misting with 5–10 mL of sterilized deionized water every 2 weeks. Individual treatments were kept in closed and transparent plastic bins and shuffled in the incubator prior to misting. After 42 weeks, each block was weighed and a sterilized chisel was used to split the block approximately in half and the inside face (average 24.22 cm^2^) of each block was assessed for the area that was colonized by *Heterobasidion* conidia. These measurements were conducted two and four weeks after cutting the blocks in half. Following assessment, each block was dried at 105 °C for 48 h to determine the final moisture content. The initial and final mass and moisture content measurements were combined to estimate the mass loss of each wood block.

### 2.6. Statistical Analysis

The field stump and laboratory wood block experiments were assessed for the likelihood of *H. occidentale* establishment and the degree of colonization on each experimental unit (stump or wood block). The likelihood of infection was analyzed with a mixed generalized linear model (glm) with stand (compartment) parameterized as a random effect and with diameter (dbh) and treatment as fixed effects. Approaching this data as a binary response avoided the complication of overdispersion in the data distribution that was introduced by the frequent lack of colonization in some treatments (i.e., zero-inflation). The stump diameter, here approximated by tree dbh, was included as a fixed effect (as opposed to a random factor) in light of recent work in Europe showing that *Phlebiopsis* treatment efficacy can increase with stump diameter [24] and it is useful to evaluate any similar effect for Sierra Nevada *Abies* forests. Furthermore, a preliminary analysis examined the effects of compartment management as fixed effects, but this was substituted for compartment-level (stand) random effects in the final model when it became clear that the management variation had no clear signal in the data, but that spatial variation between the locations was apparent. The degree of stump colonization was analyzed with an analogous analysis of variance (ANOVA) model where the compartment (stand) was parameterized as an error variable (block) with treatment and diameter as the variables of inference. For the laboratory wood block data, the same models were applied to assess the likelihood of *H. occidentale* establishment but with the block volume substituted as a subject-level random effect for each model. These models included the additional treatment timing term (initial vs. delayed treatment). Each model was assessed for adequacy of fit and the underlying assumptions of normal error distribution and homogeneous variance. The ANOVA models were assessed for normal and homogeneous error distributions; both models from the stump and laboratory experiments required a square root transformation to meet these model assumptions. We also employed the information measure AUC (area under curve) for the glm models using the criteria where AUC > 0.70 was considered adequate for inference. Dunnett’s mean separation tests were applied to determine the statistical significance of pair-wise contrasts for the treatment variable. Lastly, we examined within-treatment efficacy as measured by the within-treatment deviance from the treatment mean:

*Deviance*_trt_ = Abs (*µ*_trt_ − *y*_i_) (1)

where *µ* is the average colonization for the respective treatment (trt) and *y* is the measured colonization of each sample *i*. This deviance measure was analyzed with an identical model to the colonization ANOVA. Statistical significance was considered where *p* ≤ 0.05.

## 3. Results

Ambient *H. occidentale* inoculum loads were measured at 45.08 (range 35.68–58.49) colony forming units (CFU) m^2^ h^−1^ with a standard deviation of 8.77 CFU m^2^ h^−1^ across each of the four spore monitoring sites. The laboratory DNA assessment determined that only *H. occidentale* spores were present across the sampling locations within each of the five stands. We found no evidence of differences in ambient spore loads across stands (i.e., no statistically significant differences across stands) particularly the two stands where we performed the stump *H. occidentale* colonization prevention experiments. We measured 44.38 and 39.33 CFU m^2^ h^−1^ in compartments 310 and 670, the two stands where we conducted stump experiments, respectively. These data indicate that substantial ambient spore concentrations were present during the treatment applications despite the lack of nearby disease centers.

**Figure 1:**
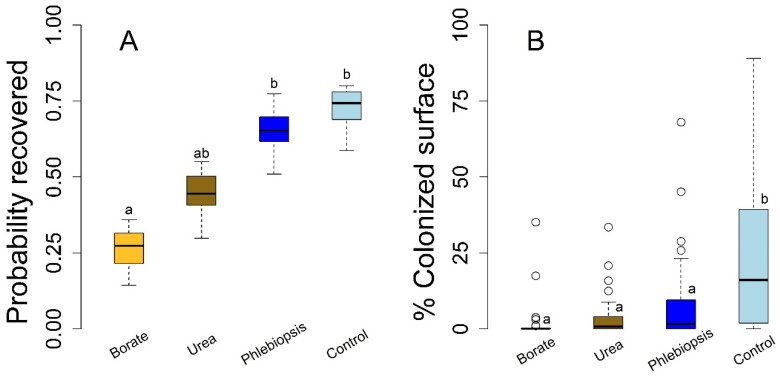
Box and whisker plots of *Heterobasidion occidentale* treatments to control pathogen establishment on *Abies concolor* stumps with the probability of pathogen recovery on a per-stump basis (pathogen colonization or infection, **A**) and the proportion of sampled disc area that was colonized by *H. occidentale* (5 cm depth, **B**). Infection probabilities were predicted values from a mixed general linear model (AUC = 0.725). Letters indicate statistically significant Dunnett’s pairwise contrasts (*p* ≤ 0.05).

Tree diameters for the stump treatment experiment averaged 56.6 cm (range 28–93.3 cm) and were not significantly different among the randomly assigned treatments. The tree diameter did not significantly influence *Heterobasidion* establishment or colonization in the stump experiments, regardless of treatment. Each stump treatment appeared successful in decreasing *H. occidentale* but success differed among treatments (Figure 1; *n* = 121). Borate application was the sole treatment with a statistically significant lower probability of *H. occidentale* establishment relative to the control and *P. gigantea* treatments (Figure 1A; *p* = 0.004 and *p* = 0.012, respectively). The probability of infection on the control stumps was 0.74 and although the infection probability was lower in all other treatments, these differences were not statistically significant for *P. gigantea* (*p* = 0.94) or the urea treatments compared to the controls (*p* = 0.17; Figure 1A). We also did not find a statistically significant difference in the probability of infection between urea and borate treatments (*p* = 0.41).

The treatment efficacy was significantly different in terms of the disc area that was colonized compared to the pathogen establishment probability. Borate, urea, and *P. gigantea* stump treatments each resulted in lower *H. occidentale* colonized area relative to the untreated controls (*p* < 0.001; Figure 1; *n* = 121). Furthermore, none of our experimental treatments were significantly different in terms of the colonized area in comparison to one another as determined by Dunnett’s pairwise contrasts. In absolute terms, the borate treatments had the lowest total colonization by *H. occidentale* and *P. gigantea* had the most colonization among the prevention treatments but the pairwise contrast between the borate and *P. gigantea* treatments was not statistically significant (*p* = 0.067). Taken as a whole, the borate treatments appeared to be the most effective to prevent *H. occidentale* infection one year after harvest, both in terms of preventing the colonization and limiting pathogen growth.

**Figure 2:**
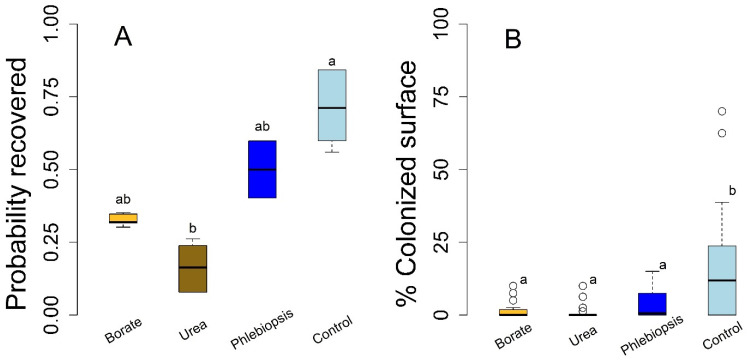
Box and whisker plots of a *Heterobasidion occidentale* control experiment on *Abies concolor* wood blocks in controlled conditions measured 42 weeks after treatment withthe probability of pathogen recovery (infection, **A**) and the proportion of the interior block surface that was colonized by *H. occidentale* (**B**). Infection probabilities were predicted values from a mixed general linear model (AUC = 0.824). Letters indicate statistically significant Dunnett’s pairwise contrasts (*p* ≤ 0.05).

Results from the laboratory wood block study largely matched those of the field stump experiment, with a few relatively small-magnitude differences (Figure 2; *n* = 96). The probability of pathogen colonization was lower in all of the treatments relative to the controls in absolute terms, but these differences were only statistically significant for the urea treatment (*p* = 0.016). We found weak evidence of differences between the borate and control treatments (*p* = 0.12) and found no evidence of differences in the probability of infection between the *P. gigantea* and control treatments (*p* = 0.48). Patterns of surface area colonization for the laboratory study were also similar to the field stump experiment; each treatment resulted in a statistically significant lower area of colonization relative to the control (*p* ≤ 0.001, each contrast). Notably, we found little evidence for efficacy differences among urea, borate, or *P. gigantea* treatments in the wood block study (*p* > 0.18, each contrast; Figure 2), a result analogous to those from the field stump experiment. In terms of both *H. occidentale* establishment and the degree of colonization, we found no evidence of differences between the delayed and immediate treatment, nor did we find evidence for treatment-delay interactions (*p* > 0.34; each interaction parameter; data not shown).

**Figure 3:**
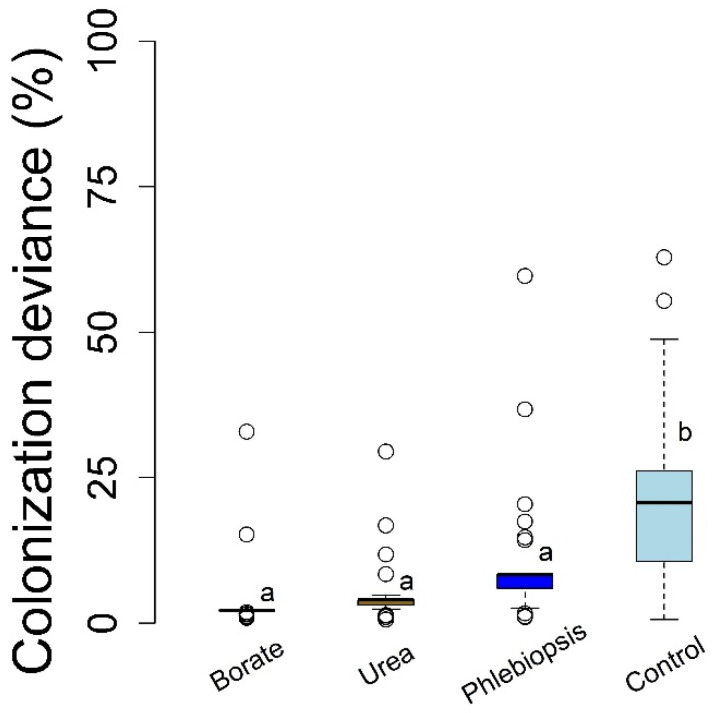
Box and whisker plot of *Heterobasidion occidentale* colonization deviance on white fir (*Abies concolor*) stumps, calculated as the absolute value of differences of each replicate from the respective treatment mean. Letters indicate statistically significant Dunnett’s pairwise contrasts (*p* < 0.05).

Colonization deviance within treatments was greatest for the control (deionized water) which was significantly higher than each of the three colonization prevention treatments (*p* < 0.0001, each contrast). Furthermore, each treatment did not result in statistically significant differences in colonization deviance between the three preventive treatments, although *P. gigantea* treatments showed an apparent trend of greater variation when compared to the borate application (*p* = 0.064). For each treatment, the 95% confidence interval of the deviance within treatments was greater than zero indicating that the treatment efficacy varied among the experimental subjects, although this deviation was much lower in each treatment compared to the control (Figure 3). Overall, we estimated treatment efficacy to reduce *H. occidentale* colonization of stumps (colonization area) between 91% and 68% for borates and *P. gigantea*, respectively, with urea treatments at an intermediate level (Table 1). Although we did find consistent trends of a somewhat lower efficacy of *P. gigantea* treatments, these were never statistically significant and each treatment clearly lowered *H. occidentale* colonization area while also reducing the variability in colonization (treatment deviance, Equation (1)) relative to the controls (Table 1).

Our experimental wounding experiment that was aimed at preventing *H. occidentale* infection was inconclusive. We did not successfully recover *H. occidentale* in any experimental tree that was included in the wounding experiment (*n* = 180). No *H. occidentale* cultures were recovered from the deionized water treated controls or positive controls that were treated with the 6.91 × 10^5^ CFU mL^−1^ solution. This inoculum solution was identical to that applied in the laboratory wood block experiment where pathogen growth was common, thus there is no basis to conclude that the pathogen was unviable in the solution. Overall, no inferences on the efficacy of borate or urea treatments in excluding *H. occidentale* establishment on the wounds of white fir were possible from our study. *Scolytus ventralis* were regularly recovered, although not abundantly, on trees included in the wounding experiment both prior to and after wounding experiments with trapping rates averaging 0.51 *S. ventralis* per study tree; however, these recoveries were not significantly different among the experimental wounding treatments (*p* > 0.5). Given the lack of successful *H. occidentale* colonization in the controls and the prevention treatments, no inferences on the potential interactions between root disease and bark beetles were possible from our study.

## 4. Discussion

Urea and borate applications are known to limit Heterobasidion root disease on stumps in the southeastern US, Canada, and Europe while *Phlebiopsis* stump treatments appear to be effective in controlling pathogen establishment in stumps in European forests [3,22,24,28]. Our findings suggest that the effects of these topical treatments may be beneficial to decrease white fir stump infection, and therefore Heterobasidion root disease, in California forests (Figure 1 and Figure 2). Our data suggest that stump infection reduction may emerge via competitive interactions that reduce pathogen growth as opposed to strictly limiting the establishment of spores on susceptible substrates (Figure 1). However, treatment may have been responsible for the frequent lack of colonization on stumps that were treated with borates and the upper bound of estimated efficacy for this treatment (101% reduction) reflects that this treatment often resulted in no colonization. Although our study quantified the efficacy of these treatments in the initial stages of pathogen establishment on stumps, the process of primary pathogen establishment on individual stumps may result in the secondary infection of multiple neighboring trees and persistent disease centers [14,15]. Previous work from outside California suggests this disease emergence process can be reduced or arrested with urea, borate, and *P. gigantea* application at the stand scale [3,22,24,28]. Thus, it is likely that the treatments that were the focus of this study will yield benefits in terms of reduced disease impacts, tree mortality, growth, or wood defects on neighboring trees in white fir forests. In other words, tree-level treatments could provide benefits at the stand level over the longer term, a prospect that calls for further study [19,22,24]. Given the low cost, minimal potential for environmental impact, and wide availability, the broader use of these treatments to limit disease establishment in California white fir stands and their associated silvicultural systems is likely to be worthwhile for many managers. However, *P. gigantea* products, such as the Rotstop^®^C formulation (BioFOrest Tehnologies Inc., Sault Ste. Marie, Canada) that is commercially available in the US, are based on an eastern strain of *P. gigantea*, are genetically distinct from western strains of the same fungus and recent work has argued against deploying them in western US forests [47]. Our study was the first to apply and report the efficacy of California isolates of *P. gigantea* in terms of preventing *H. occidentale* establishment or growth on stumps and is thus extremely timely and provides data on a possible future alternative to the commercially available *P. gigantea* formulation.

Both the field and laboratory experiments suggest that the biocontrol may come with some decrease in terms of efficacy which deserves attention before it is broadly adopted. For example, borate and urea treatments both appeared to limit *H. occidentale* establishment in the field (stump) and laboratory studies, respectively, while *Phlebiopsis* did not in either setting (Figure 1 and Figure 2). Rather, *H. occidentale* was a common co-occurring fungus with *Phlebiopsis*, although *H. occidentale* growth was significantly reduced by *Phlebiopsis* treatment in both the field and laboratory experimental settings. The urea and borate treatments appeared to prevent *H. occidentale* establishment more effectively than *P. gigantea*, while all three treatments reduced pathogen growth and its deviance among the study subjects. Together, these results indicate that all treatments, including that with *P. gigantea* have a broad applicability. That is, they are likely to be effective, independent of the unknown sources of variation in efficacy across subjects (presumably microclimate or host genotypic differences) (Figure 3). In a study similar, borate, urea, and *Phlebiopsis* efficacy to prevent *Heterobasidion* establishment on Norway spruce (*P. abies*) stumps in Europe which used higher urea concentrations (>20%), commercial *Phlebiopsis* products (Rotstop^®^_,_ Lallemand Finland Oy Verdera, Kurjenkellontie, Finland), and borate powder showed efficacy that was comparable to our results [28]. The differences between our study and previous work could be driven by differences in host, pathogen, biocontrol strain (including cryptic species), or the environment between the two studies. Additional trials could help resolve this apparent discrepancy and determine if California *P. gigantea* strains are, in fact, less effective or if the concentration or application rate can be adjusted to reach similar levels of efficacy. It is also possible that *P. gigantea* efficacy is best estimated over a longer period given that a living organism is likely to be active in a stump much longer than the chemical treatments (see [29]). Furthermore, this study alone cannot determine if the difference in colonization vs. pathogen growth (Figure 1) will result in long term and stand level differences in disease emergence, although this stand-level treatment benefit has been empirically demonstrated in several experiments conducted in Europe [24,27,29].

Overall, each treatment performed comparably to those reported in similar experiments that were conducted in other host systems to control Heterobasidion root and butt rot disease, even though the concentrations of our urea and borate applications were lower than those used in other experiments [22,27,28]. It is possible that higher solution concentrations could increase the treatment efficacy in white fir forests of California and any such follow-up would improve management by integrating a broader set of seasonality. Additionally, we caution that seasonality may also have a significant impact on the efficacy of the treatment [23], and thus the comparative efficacy of treatments that are performed in different seasons in California should also be investigated to gain a full understanding of the potential to prevent this disease. Our measured ambient spore levels (45.1 ± 8.77 CFU m^2^ h^−1^) exceeded those that are generally expected to result in Heterobasidion root disease and are typical of those measured at distances greater than 100 m from disease centers [40,42,48]. Our study was intentionally designed to estimate efficacy in production forestry situations where *Heterobasidion* pathogen inoculum is known to be high. Ambient *Heterobasidion* inoculum can be much higher in close proximity to disease centers in California and elsewhere [40,41,42], suggesting that treatment tests across inoculum gradients would also be wise before applying these treatments outside of the conditions we evaluated here. Regardless, our data show clear potential benefits of each stump treatment during autumnal stand harvest in areas where Heterobasidion root disease is a management problem. Furthermore, given the low cost and wide availability of urea and borate compounds, these can be rapidly adopted in stand management in accordance with state and local regulations once they are shown to be effective at the stand scale.

*Heterobasidion occidentale* conidiophores were not observed in any of the wood core samples that were extracted from the treated wounded trees, including the positive and negative controls. Stumps can be artificially infected with conidia [22,49] but artificially infecting true fir wounds is a novel experimental challenge that may require a different experimental approach, perhaps by using basidiospores. Preventing wound infection may be pivotal in controlling Heterobasidion root disease in true firs in California and in Western forests with a true fir component given that field studies in conjunction with population genetics analyses suggest *H. occidentale* spread in Western *Abies* species may be more common through establishment of the pathogen in wounds compared to stumps [3,14,19]. Regrettably, our lack of success when attempting to infect wounds using conidia limits the value of this part of our experiment. The lack of pathogen establishment in standing trees also limits our inferences into pathogen-insect interactions; we frequently recovered *S. ventralis* on the trees monitored as part of our wounding experiment, but we found no evidence that insect landing was associated with specific treatments, and as we had no successful recoveries of *H. occidentale* on any of our wound experiment trees, we can offer no further insight into these potential interactions. We also must acknowledge that the physiological effects of *Heterobasidion* infection on a tree may take much longer than one year to be measurable in terms declining vigor that, in turn, may be responsible for beetle attraction [11]. In support of this speculation, recent work on *Armillaria* species, a long-lived forest root pathogen showed evidence of root disease–bark beetle associations, particularly over multiple years with variable climate stressors (drought and heat [38]).

## 5. Conclusions

*Heterobasidion occidentale* causes long-lasting disease impacts which can create a serious management challenge in California fir forests. Although our wound experiment yielded no insights, we found that urea, borate, and two locally collected strains of *P. gigantea* were effective in limiting the pathogen colonization of stumps in a field experiment that was conducted under conditions that were typical of an autumnal forest harvest. These patterns were confirmed by a paired laboratory experiment that was conducted on wood blocks. Our results suggest that low-cost chemical control, through urea or borate treatments to stumps may reduce subsequent disease emergence following forest operations and argue for testing this expectation at the stand scale. Our results also suggest potential for the development of a *P. gigantea* product using California-based strains that are similar to products that are developed in Europe and in the Southeastern United States. Given the potential for *H. occidentale* to impact stand growth and tree mortality over multiple decades, effective treatments are likely to yield benefits in terms of timber production and forest sustainability in mid-elevation Sierra Nevada forests, and perhaps more broadly across the west. These forests provide highly valuable timber, recreation, regional carbon sequestration benefits, and are central to issues related to wildfire dynamics and impacts. In light of these resources and resource issues, it is clear that inexpensive and effective Heterobasidion root disease treatments could have great practical value for the region.

## Figures and Tables

**Table 1:** Mean and 95% confidence intervals (CI) for the within-treatment colonization deviance and estimated efficacy (% difference relative to control) as estimated by differences in the area that was colonized by *H. occidentale* for three stump-infection prevention treatments.

Treatment	Mean	Upper 95% CI	Lower 95% CI
	Within-treatment deviance (%)		
**Borate**	3.48	5.65	1.31
***P. gigantea***	10.06	13.74	6.39
**Urea**	5.0	6.94	3.06
**Control**	22.68	29.01	16.35
	Treatment efficacy relative to control (%)		
**Borate**	91.65	101.27	82.03
***P. gigantea***	68.08	87.31	48.86
**Urea**	84.65	94.75	74.54

## Data Availability

Not applicable.
